# No Change in Bicarbonate Transport but Tight-Junction Formation Is Delayed by Fluoride in a Novel Ameloblast Model

**DOI:** 10.3389/fphys.2017.00940

**Published:** 2017-12-06

**Authors:** Róbert Rácz, Anna Földes, Erzsébet Bori, Ákos Zsembery, Hidemitsu Harada, Martin C. Steward, Pamela DenBesten, Antonius L. J. J. Bronckers, Gábor Gerber, Gábor Varga

**Affiliations:** ^1^Department of Oral Biology, Semmelweis University, Budapest, Hungary; ^2^Department of Anatomy, Iwate Medical University, Iwate, Japan; ^3^School of Medical Sciences, University of Manchester, Manchester, United Kingdom; ^4^Department of Orofacial Sciences, University of California, San Francisco, San Francisco, CA, United States; ^5^Department of Oral Cell Biology, Academic Centre for Dentistry Amsterdam (ACTA), Amsterdam, Netherlands; ^6^Department of Anatomy, Histology and Embryology, Semmelweis University, Budapest, Hungary

**Keywords:** HAT-7, ameloblast, bicarbonate, ion transport, pH regulation, fluoride, tight-junction, transepithelial resistance

## Abstract

We have recently developed a novel *in vitro* model using HAT-7 rat ameloblast cells to functionally study epithelial ion transport during amelogenesis. Our present aims were to identify key transporters of bicarbonate in HAT-7 cells and also to examine the effects of fluoride exposure on vectorial bicarbonate transport, cell viability, and the development of transepithelial resistance. To obtain monolayers, the HAT-7 cells were cultured on Transwell permeable filters. We monitored transepithelial resistance (TER) as an indicator of tight junction formation and polarization. We evaluated intracellular pH changes by microfluorometry using the fluorescent indicator BCECF. Activities of ion transporters were tested by withdrawal of various ions from the bathing medium, by using transporter specific inhibitors, and by activation of transporters with forskolin and ATP. Cell survival was estimated by alamarBlue assay. Changes in gene expression were monitored by qPCR. We identified the activity of several ion transporters, NBCe1, NHE1, NKCC1, and AE2, which are involved in intracellular pH regulation and vectorial bicarbonate and chloride transport. Bicarbonate secretion by HAT-7 cells was not affected by acute fluoride exposure over a wide range of concentrations. However, tight-junction formation was inhibited by 1 mM fluoride, a concentration which did not substantially reduce cell viability, suggesting an effect of fluoride on paracellular permeability and tight-junction formation. Cell viability was only reduced by prolonged exposure to fluoride concentrations greater than 1 mM. In conclusion, cultured HAT-7 cells are functionally polarized and are able to transport bicarbonate ions from the basolateral to the apical fluid spaces. Exposure to 1 mM fluoride has little effect on bicarbonate secretion or cell viability but delays tight-junction formation, suggesting a novel mechanism that may contribute to dental fluorosis.

## Introduction

Dental enamel is the hardest material in the human body and its mineral concentration is also the highest. Its major disorders result from either environmental or genetic conditions. In both cases mineral formation can be greatly impaired. Also dental caries and erosion are important enamel-loss conditions where reconstruction would be the optimal solution. Ameloblasts secrete enamel in a two-stage process. First a slightly mineralized matrix structure is built. Then the remodeling of this matrix results in a high level of mineralization (Robinson, 2014). Ameloblasts have epithelial tight junctions which close the intercellular space allowing the preservation of great concentration gradients between the apical and basal sides of the cells. Calcium and phosphate ions are actively transported into the mineralization space by an only partially understood process.

Acid/base balance is crucial during enamel hydroxyapatite formation since the crystal growth depends upon a delicate cellular control of the ionic composition and pH of the extracellular fluid (Takagi et al., 1998). Hydroxyapatite formation during the maturation stage of amelogenesis liberates an enormous quantity of protons. Thus, sustained crystal growth requires these protons to be neutralized (Smith, 1998; Josephsen et al., 2010; Lacruz et al., 2010) by bicarbonate transported directly into the enamel space. The available information about electrolyte transport by ameloblasts is based almost exclusively on expressional studies, immunohistochemistry, and chemical composition analysis, with little functional support (Schroeder and Listgarten, 1997; Bosshardt and Lang, 2005). Consequently, the mechanistic models have hitherto been purely hypothetical.

We have therefore developed an *in vitro* model, using the HAT-7 rat ameloblast cell line, to study epithelial ion transport during amelogenesis (Bori et al., 2016). HAT-7 is a dental epithelial cell line derived from the cervical loop epithelium of a rat incisor (Kawano et al., 2002). Immunocytochemical studies have shown that HAT-7 cells exhibit several ameloblast characteristics, including the expression of amelogenin and ameloblastin (Kawano et al., 2002) and also maturation-stage ameloblast markers such as kallikrein-4 (Klk4) and amelotin. We have to note, however that further studies are needed to determine how well HAT-7 cells could serve as an optimal model for maturation ameloblast function. In our preliminary, proof-of-concept work (Bori et al., 2016) we demonstrated that our 2D *in vitro* model is suitable for functional investigations of pH regulation, mineral transport, and tight-junction formation. Confluent monolayers of HAT-7 cells grown on permeable supports are functionally polarized, they express ion transporters and tight-junction proteins and they mediate vectorial ${\text{HCO}}_{3}^{-}$ transport.

Enamel fluorosis is a developmental disturbance caused by intake of supraoptimal levels of fluoride during early childhood (Aoba and Fejerskov, 2002; Denbesten and Li, 2011). The enamel defects consist of horizontal thin white lines, opacities (subsurface porosities), discolorations, and pits of various sizes. The molecular mechanism underlying enamel fluorosis is still unknown. Possible explanations include direct toxic effects of fluoride on ameloblasts, fluoride-related alterations in the forming enamel matrix, reduced proteolytic activity due to fluoride incorporation into growing enamel crystals, the potential effects of fluoride on matrix pH, and incomplete barrier formation at the mineralization front (Aoba and Fejerskov, 2002; Denbesten and Li, 2011; Lyaruu et al., 2014). None of these hypotheses can be directly proved because there is a lack of appropriate experimental models.

Our newly developed HAT-7 ameloblast monolayer model (Bori et al., 2016) may offer a reasonable basis for such studies. We can hypothesize that fluorosis is due to a combination of direct cytotoxic effects causing cell death, the delayed development of tight junctions, which are necessary to form a sealed barrier between apical and basolateral surfaces, and a direct inhibitory effect of fluoride on vectorial calcium and/or bicarbonate transport. The purpose of the present study was (1) to identify the basolateral acid/base transporters affecting intracellular pH regulation in our polarized HAT-7 cell model, (2) to assess whether acute fluoride exposure disturbs transepithelial ${\text{HCO}}_{3}^{-}$ secretion in this model, and (3) to assess viability, development of transepithelial resistance, and gene expression of tight-junction proteins of polarized HAT-7 cells in the presence of fluoride.

## Materials and methods

### Cell culture

To obtain polarized monolayers (Bori et al., 2016), HAT-7 cells were seeded on permeable polyester Transwell culture inserts with 0.4 μm pore size and 1.12 cm^2^ surface area (Costar, Corning, NY, USA) and were cultured in DMEM/F12 Ham medium (Sigma-Aldrich, St. Louis, MO, USA) supplemented with 10% HyClone fetal bovine serum (Thermo Scientific, Waltham, MA, USA), 100 U/ml penicillin, 10 μg/ml streptomycin (Sigma), CaCl_2_ (2.1 mM final concentration), and 10^−5^ mM dexamethasone (Sigma) (Arakaki et al., 2012) as described previously (Bori et al., 2016). They were grown in a humidified atmosphere containing 5% CO_2_ at 37°C.

### Measurement of transepithelial electrical resistance

Transepithelial electrical resistance (TER) values of HAT-7 cells grown on Transwell membranes incubated in 12-well plates were measured using an epithelial volt-ohmmeter (EVOM, World Precision Instruments, Hamden CT, USA) on 5 consecutive days prior to microfluorometric measurements or during NaF treatments. TER values give an indication of the paracellular permeability to electrolytes, and thus tight-junction formation, which are key characteristics of secretory and absorptive epithelia. In multi-day fluoride exposure experiments, 24 h after cell seeding on Transwells, the medium was changed to 0 (control), 0.3, 0.6, or 1 mM NaF-containing medium.

### Microfluorometry

Intracellular pH (pH_i_) in HAT-7 cells was measured by microfluorometry as described previously (Szucs et al., 2006; Bori et al., 2016). Briefly, the cells were loaded with a fluorescent dye, BCECF-AM, that is sensitive to intracellular pH and therefore capable of indirectly measuring H^+^ and/or ${\text{HCO}}_{3}^{-}$ movements through the cell membrane. Particular elements of ${\text{HCO}}_{3}^{-}$ transport can be identified by modifying the extracellular environment (e.g., specific ion withdrawal, application of transporter inhibitors).

Cells grown on Transwell membranes were mounted in a minichamber on a Nikon Eclipse TE200 inverted fluorescence microscope and were bilaterally superfused at 3 ml/min. Illumination was alternated between 490 and 440 nm excitation wavelengths. Fluorescence was measured every 5 s at 530 nm using a photomultiplier tube and amplifier (Cairn Research, Faversham, Kent, UK) and data were acquired using DASYLab software (Measurement Computing, Norton, MA). Fluorescence data were corrected for autofluorescence. Using calibration data obtained with the nigericin/high potassium method (Thomas et al., 1979) the ratio of fluorescence signals at the two excitation wavelengths was converted to pH_i_.

The following solutions were used for perfusion: standard HEPES-buffered solution containing (in mM) 137 NaCl, 5 KCl, 1 CaCl_2_, 1 MgCl_2_, 10 D-glucose, and 10 HEPES (4-(2-hydroxyethyl)-1-piperazineethanesulfonic acid), equilibrated with 100% O_2_; standard ${\text{HCO}}_{3}^{-}$-containing HEPES-buffered solution containing (in mM) 116 NaCl, 25 NaHCO_3_, 5 KCl, 1 CaCl_2_, 1 MgCl_2_, 10 D-glucose, and 5 HEPES, equilibrated with 5% CO_2_/95% O_2_. For Na^+^ withdrawal, Na^+^ was replaced by equimolar N-methyl-D-glucamine (NMDG). For Cl^−^ withdrawal, Cl^−^ was replaced with equimolar gluconate. All solutions were adjusted to pH 7.4 at 37°C. For inhibiting specific transport processes 100 μM DIDS was used to block anion exchangers, 300 μM amiloride to block Na^+^-H^+^ exchange, 500 μM H_2_DIDS for Na^+^-${\text{HCO}}_{3}^{-}$ cotransport, and 100 μM bumetanide for NKCC. For stimulation of transport, 50 μM ATP was used to elevate intracellular calcium concentrations and 10 μM forskolin, in combination with 500 μM IBMX (3-isobutyl-1-methylxanthine), was used to elevate intracellular cAMP levels. All reagents were purchased from Sigma (Sigma-Aldrich, St. Louis, MO, USA), except H_2_DIDS and BCECF-AM (both from Molecular Probes, Eugene, OR, USA) and NaF (Molar Chemicals, Hungary).

### Cell viability assays

Cell viability was tested by alamarBlue assay (Thermo Scientific, Waltham, MA, USA) according to the manufacturer's protocol. Cells (10^4^ per well) were plated in 96-well plates, and experiments started 24 h after plating. At this time the medium was supplemented with various concentrations of NaF. After 48 and 96-h exposures to fluoride, the cells' metabolic activity was evaluated by measuring the alamarBlue fluorescence at 590 nm (with excitation at 560 nm) using a Perkin-Elmer LS50B luminescence spectrometer. Each treatment was applied in six parallel wells.

### Quantitative PCR

The expression of tight-junction forming genes was estimated by quantitative RT-PCR as described previously (Hegyesi et al., 2015; Bori et al., 2016). Total RNA was isolated 3 days after seeding from Transwell samples incubated in medium containing 0, 0.6, and 1 mM NaF, by GeneJET RNA Purification Kit (Thermo Scientific, Waltham, MA, USA). Approximately 1–2 μg of total RNA was reverse transcribed by Maxima First Strand cDNA Synthesis Kit for RT-qPCR (Thermo Scientific, Waltham, MA, USA). The cDNA was then used in quantitative PCR reactions. qPCR amplification was performed using the ABI StepOne System with TaqMan Universal Master Mix II and predesigned primers Tjp1: Rn02116071; Cldn1: Rn00581740; Cldn4: Rn01196224; Cldn8: Rn01767199; Cldn16: Rn00590884; and Cldn19: Rn01416537 (Applied Biosystems, Foster City, CA, USA). Acidic ribosomal protein P0 (Rplpo: Rn00821065) was used as internal control and the ΔΔCt method was used to quantify gene expression with ABIPrism 2.3 software. Each sample was measured in three biological replicates and in three technical parallels.

### Statistical analysis

Data are presented as mean ± SEM. Statistical analyses were performed using one-way or repeated-measures ANOVA, followed by Dunnett's *post-hoc* test. Unpaired *t*-tests were applied when only two groups were to be compared. As transepithelial resistance experiments resulted in large differences in SEM values, thus not permitting parametric tests, the non-parametric Kruskal-Wallis test and Dunn's *post-hoc* test were used to compare TER values.

## Results

### Evidence for activity of the major basolateral transporters participating in intracellular pH regulation in HAT-7 cells

In our previous work we showed data suggesting the existence of vectorial, basolateral-to-apical bicarbonate transport in HAT-7 ameloblast cells but we did not identify the individual transporters at the basolateral side (Bori et al., 2016).

#### Na^+^-H^+^ exchanger activity at the basolateral membrane

**Figure 1:**
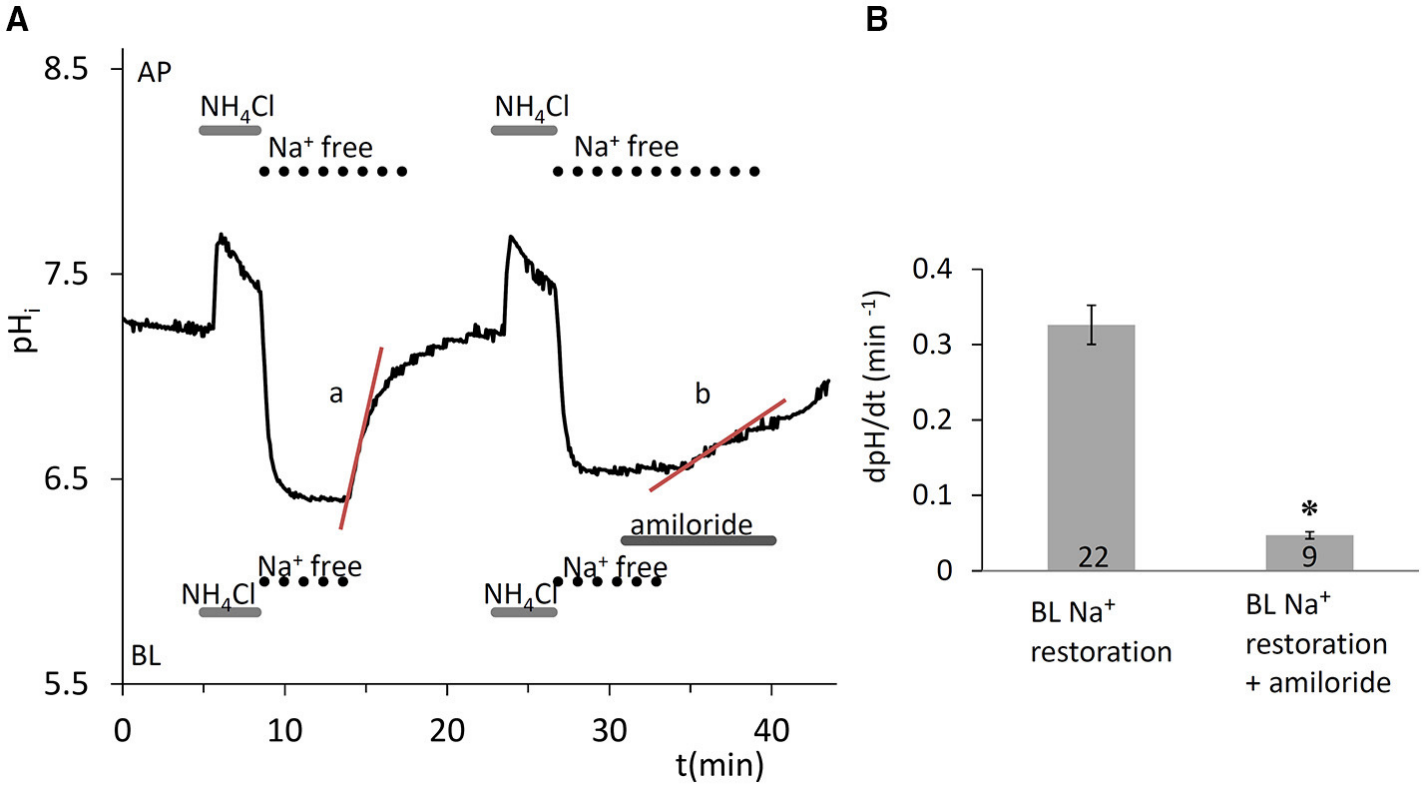
Recovery of pH_i_ in HAT-7 cells exposed to an acid load in the absence of ${\text{HCO}}_{3}^{-}$/CO_2_. **(A)** HAT-7 cells grown on Transwell membranes were exposed bilaterally to 20 mM NH_4_Cl followed by bilateral substitution of Na^+^ with NMDG^+^. Recovery of pH_i_ (a) can be seen following basolateral restoration of external Na^+^. Inhibition of pH_i_ recovery (b) can be seen following restoration of Na^+^ in the presence of basolateral (BL) amiloride (300 μM) in order to selectively block the NHE1 exchanger. **(B)** Mean dpH/dt (± SEM) values were calculated from the initial rates of increase in pH_i_ following restoration of Na^+^ in the presence and absence of the inhibitor (*n* = 9–22). ^*^p < 0.05 compared with control.

The ammonium pulse technique (Boron and De Weer, 1976) was used to induce intracellular acidification, and the rate of recovery of pH_i_ from the acid load was measured in the absence of ${\text{HCO}}_{3}^{-}$/CO_2_. Removal of Na^+^ from both sides of the epithelium after the ${\text{NH}}_{4}^{+}$ pulse completely blocked the recovery of pH_i_ from the acidification (Figure 1A), indicating the Na^+^ dependence of the transporters responsible for pH_i_ regulation. Na^+^ restoration on the basolateral side caused a rapid recovery of pH_i_ which was sensitive to 300 μM amiloride (Figures 1A,B) indicating the existence of basolateral Na^+^-H^+^ exchanger (NHE) activity, most probably due to NHE1 which is ubiquitously expressed at the basolateral membrane of secretory epithelia.

#### Na^+^-${\text{HCO}}_{3}^{-}$ cotransporter activity at the basolateral membrane

**Figure 2:**
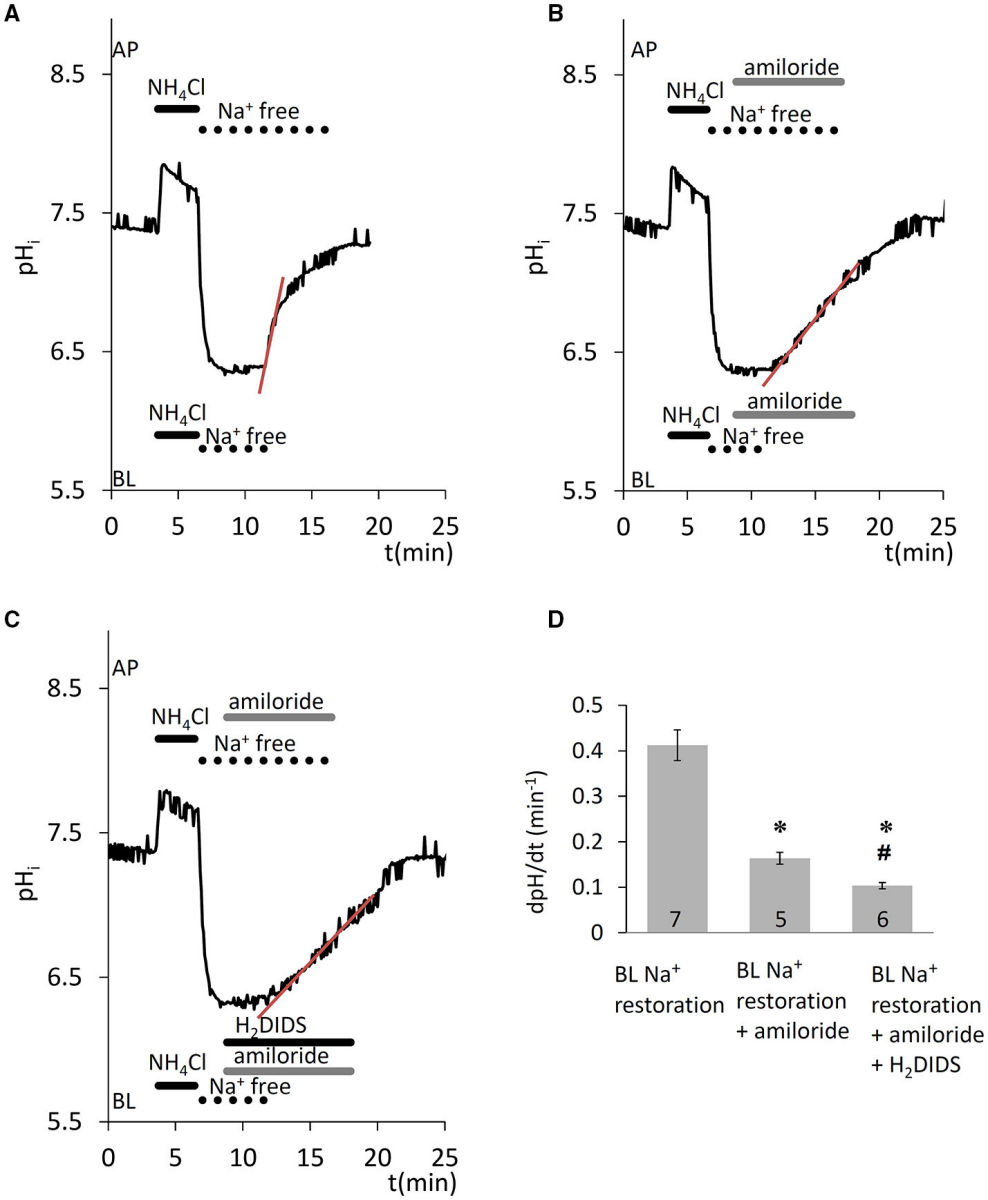
Recovery of pH_i_ in HAT-7 cells exposed to an acid load in the presence of ${\text{HCO}}_{3}^{-}$/CO_2_. HAT-7 cells grown on Transwell membranes were exposed bilaterally to 20 mM NH_4_Cl followed by bilateral substitution of Na^+^ with NMDG^+^. **(A)** Recovery of pHi following basolateral restoration of extracellular Na^+^. **(B)** Inhibition of pH_i_ recovery following restoration of Na^+^ in the presence of basolateral (BL) amiloride (300 μM). **(C)** As for panel B but with the amiloride-containing BL solution supplemented with H_2_DIDS (500 μM) in order to block the NBCe1 cotransporter. **(D)** Mean dpH/dt ± SEM values calculated from the initial rates of increase in pH_i_ following restoration of Na^+^ in the presence and absence of the inhibitors (*n* = 5–7). ^*^p < 0.05 compared to control, #*p* < 0.05 compared to amiloride alone.

In the presence of ${\text{HCO}}_{3}^{-}$/CO_2_, removal of Na^+^ from both sides, after acid load, blocked the recovery of pH_i_ (Figure 2A), suggesting that the ${\text{HCO}}_{3}^{-}$ transporters involved in pH_i_ regulation are also Na^+^ dependent and thus likely to include the Na^+^-${\text{HCO}}_{3}^{-}$ cotransporter NBCe1. Na^+^ restoration on the basolateral side caused a sharp increase in pH_i_, which was partially amiloride sensitive (Figures 2B,D, *p* < 0.05 vs. control) and therefore only partially attributable to NHE1. Additionally, when the NBCe1 inhibitor H_2_DIDS (500 μM) was applied in addition to amiloride, further significant inhibition of pH_i_ recovery was observed (Figures 2C,D, *p* < 0.05 vs. amiloride given alone). Thus, the pH_i_ regulatory mechanisms following intracellular acidification seem to involve both ${\text{HCO}}_{3}^{-}$ uptake by NBCe1, and H^+^ extrusion by NHE1 in HAT-7 cells.

#### Na^+^-K^+^-2Cl^−^ cotransporter activity at the basolateral membrane

**Figure 3:**
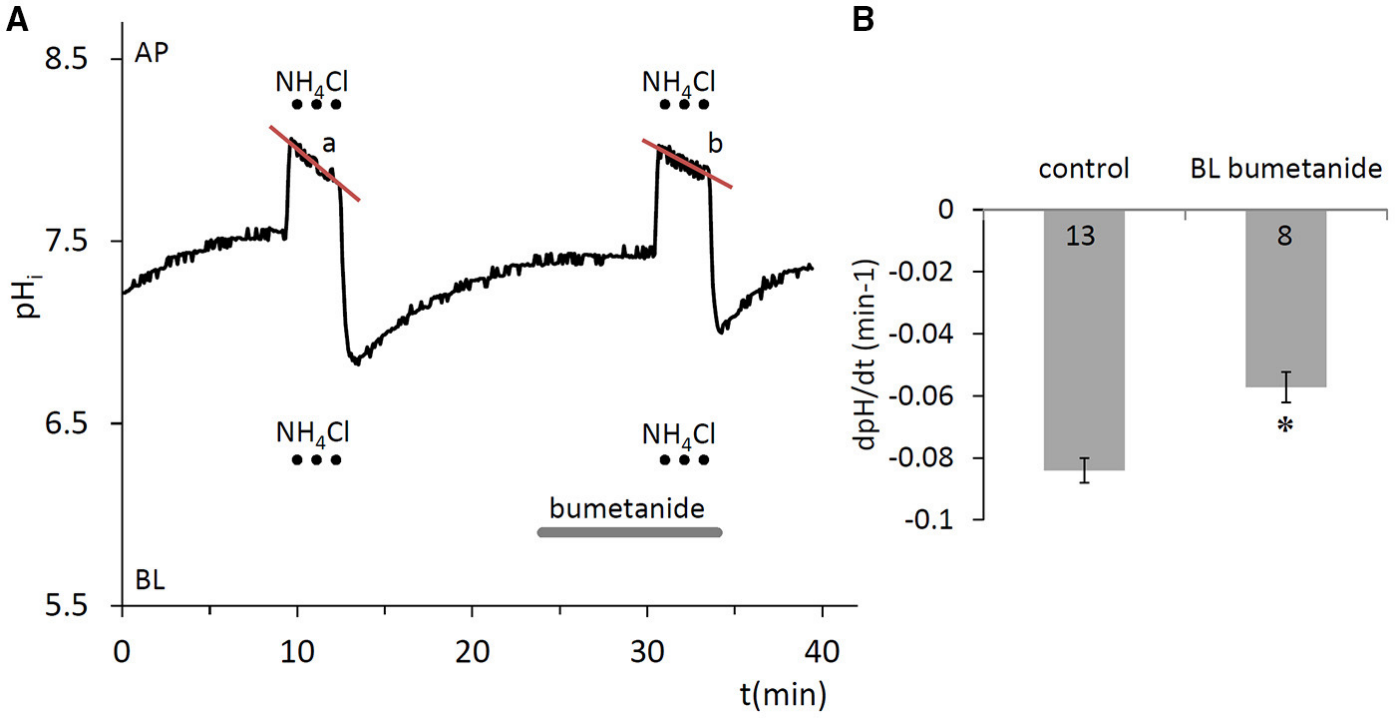
Compensation of pH_i_ change in HAT-7 cells exposed to an alkali load in the absence of ${\text{HCO}}_{3}^{-}$/CO_2_. **(A)** HAT-7 cells grown on Transwell membranes were exposed bilaterally to 20 mM NH_4_Cl, during which time partial pHi compensation (a) was observed. Inhibition of pH_i_ compensation can be seen (b) in the presence of basolateral (BL) bumetanide (100 μM), a selective blocker of the NKCC1 cotransporter. **(B)** Mean dpH/dt ± SEM values were calculated from the rate of pH_i_ decrease during NH_4_Cl exposure in the presence and absence of the inhibitor (*n* = 8–13). ^*^p < 0.05 compared to control.

A potentially important factor that may contribute to the partial recovery of pH_i_ from the alkalinization that occurs during the ${\text{NH}}_{4}^{+}$ pulse is the acidifying effect of ${\text{NH}}_{4}^{+}$ uptake. This could be mediated by the Na^+^-K^+^-2Cl^−^ cotransporter (NKCC1) which is known to transport ${\text{NH}}_{4}^{+}$ in place of K^+^ (Paulais and Turner, 1992b). Basolateral application of the NKCC1 inhibitor bumetanide (100 μM) (Shumaker and Soleimani, 1999), significantly slowed the acidification that occurred during the ${\text{NH}}_{4}^{+}$ pulse (*p* < 0.05, Figure 3). This suggests that NKCC1 is present in HAT-7 cells and is consistent with our previous RT-PCR data (Bori et al., 2016).

#### Anion-exchanger activity at the basolateral membrane

**Figure 4:**
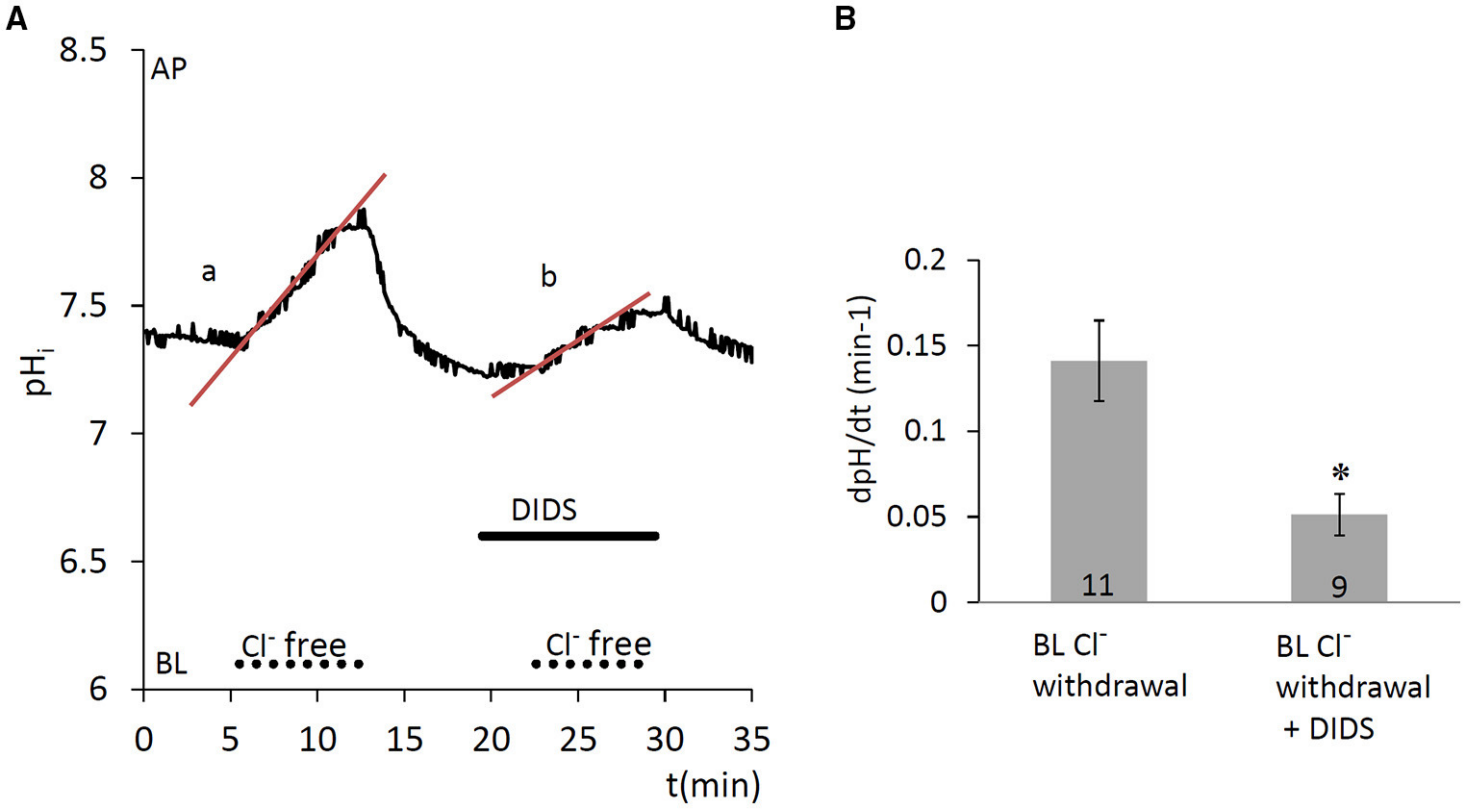
Increase in pH_i_ in HAT-7 cells upon Cl^−^ withdrawal in the presence of ${\text{HCO}}_{3}^{-}$/CO_2_. **(A)** HAT-7 cells grown on Transwell membranes were exposed basolaterally to Cl^−^-free ${\text{HCO}}_{3}^{-}$-containing HEPES solution. An increase in pH_i_ (a) can be seen, most probably as a result of ${\text{HCO}}_{3}^{-}$ influx. Upon restoration of basolateral Cl^−^, pH_i_ recovers to the baseline. Basolateral administration of DIDS (100 μM) prior to a second Cl^−^ withdrawal inhibited the increase in pH_i_ (b) suggesting the presence of a ${\text{HCO}}_{3}^{-}$/Cl^−^ exchanger. **(B)** Mean dpH/dt (± SEM) values were calculated from the initial rates of increase in pH_i_ following removal Cl^−^ in the presence and absence of the inhibitor (*n* = 9–11). ^*^p < 0.05 compared to control.

Since anion secretion by ameloblasts involves Cl^−^/${\text{HCO}}_{3}^{-}$ exchange at the basolateral membrane (Lyaruu et al., 2008), the next series of experiments was designed to test the activity of anion exchangers in HAT-7 cells. Extracellular Cl^−^ was substituted with a non-transported anion, gluconate, and the resulting change in pH_i_ was recorded. Substitution of Cl^−^ reverses the normal concentration gradient for Cl^−^. If anion exchangers are present, the resulting efflux of Cl^−^ will be coupled to a rapid uptake of ${\text{HCO}}_{3}^{-}$ and this will result in a measurable increase in pH_i_. Indeed, removal of basolateral Cl^−^ from the HEPES-buffered bath solution elicited an increase in pH_i_ (Figure 4), likely due to ${\text{HCO}}_{3}^{-}$ influx, which was significantly inhibited by the anion exchange inhibitor DIDS (100 μM) (*p* < 0.05 vs. control). This suggests that a DIDS-sensitive anion exchanger, most probably AE2, is present at the basolateral membrane of HAT-7 ameloblast cells.

### Lack of effect of acute fluoride exposure on bicarbonate secretion in HAT-7 cells

**Figure 5:**
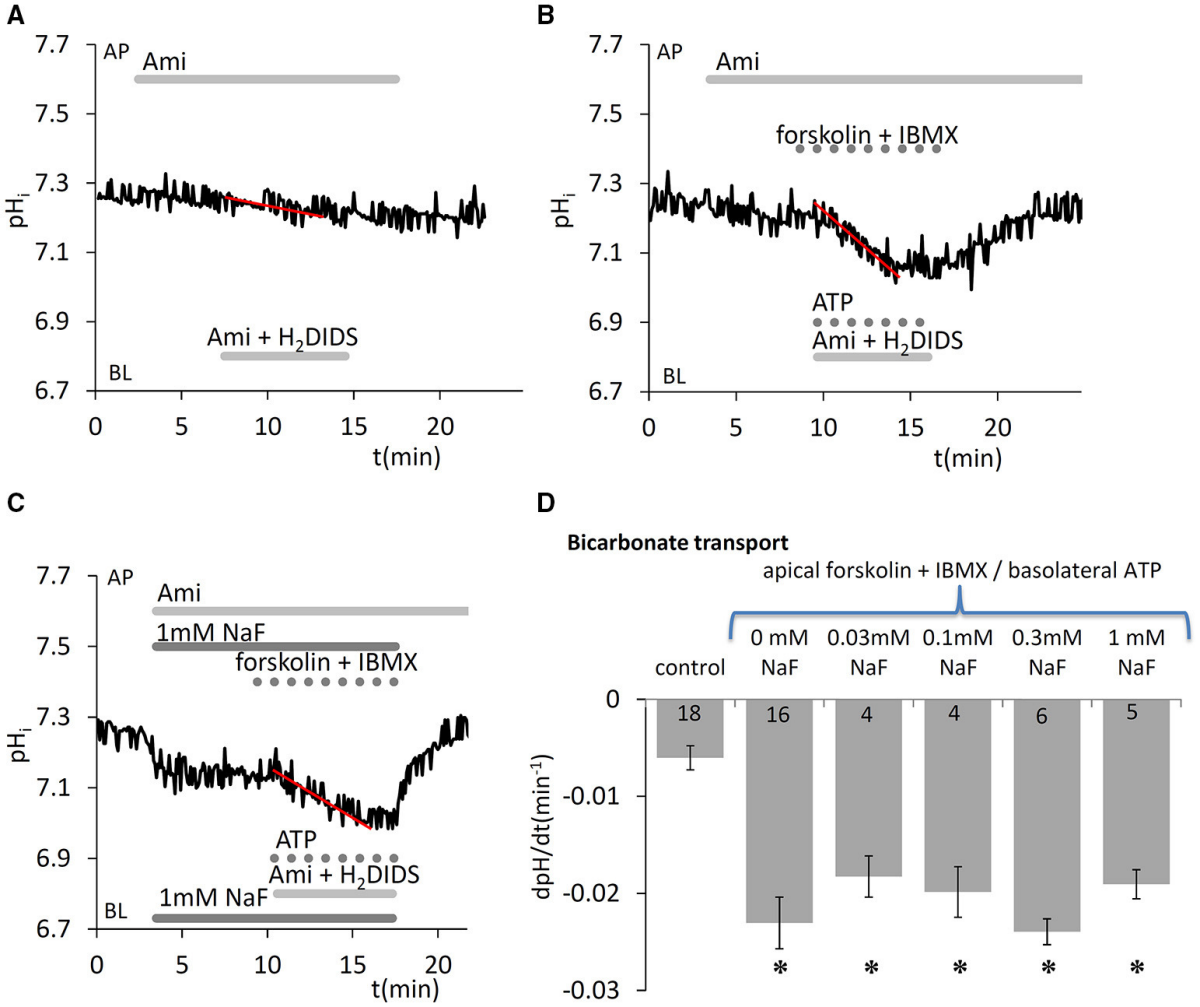
Effects of fluoride on ${\text{HCO}}_{3}^{-}$ secretion by HAT-7 cells stimulated with ATP and forskolin. Basolateral ${\text{HCO}}_{3}^{-}$ uptake in HAT-7 cells grown on Transwell membranes was inhibited by simultaneous basolateral (BL) application of 500 μM H2DIDS and 300 μM amiloride (Ami). Amiloride was also included in the apical (AP) perfusate to inhibit any apical NHE activity. Representative pH_i_ traces obtained **(A)** in unstimulated control conditions, **(B)** in cells stimulated with ATP (50 μM), forskolin (10 μM), and IBMX (500 μM), and **(C)** in stimulated cells exposed to 1 mM NaF. **(D)** Mean dpH/dt ± SEM values calculated from the initial rates of decrease in pH_i_ in unstimulated cells (control) and cells stimulated with ATP, forskolin and IBMX, and pretreated with a range of NaF concentrations. ^*^p < 0.05 compared with control.

Besides the cotransport of ${\text{HCO}}_{3}^{-}$ through the basolateral membrane by NBCe1 (using the Na^+^ gradient as a driving force), cells can also accumulate ${\text{HCO}}_{3}^{-}$ by the diffusion of CO_2_ into the cells, its conversion to ${\text{HCO}}_{3}^{-}$ and H^+^ by carbonic anhydrases, and subsequent H^+^ extrusion by NHE1. We demonstrated in our previous paper that when ${\text{HCO}}_{3}^{-}$ uptake is blocked on the basolateral side by NBCe1 and NHE1 inhibitors, the continuing apical efflux of ${\text{HCO}}_{3}^{-}$ leads to a slow intracellular acidification. This can be further enhanced by simultaneous application of Ca^2+^- and cAMP-mobilizing stimuli (ATP and forskolin/IBMX, respectively) (Bori et al., 2016). In the present work we measured this initial acidification rate, an index of ${\text{HCO}}_{3}^{-}$ secretion, to test whether acute NaF exposure has any effect on vectorial ${\text{HCO}}_{3}^{-}$ transport. We found that fluoride in the concentration range 0.03–1.0 mM did not affect ${\text{HCO}}_{3}^{-}$ secretion evoked by simultaneous stimulation with 50 μM ATP, 10 μM forskolin, and 500 μM IBMX (Figure 5).

### Development of transepithelial resistance, cell viability, and gene expression in HAT-7 cells exposed to fluoride

**Figure 6:**
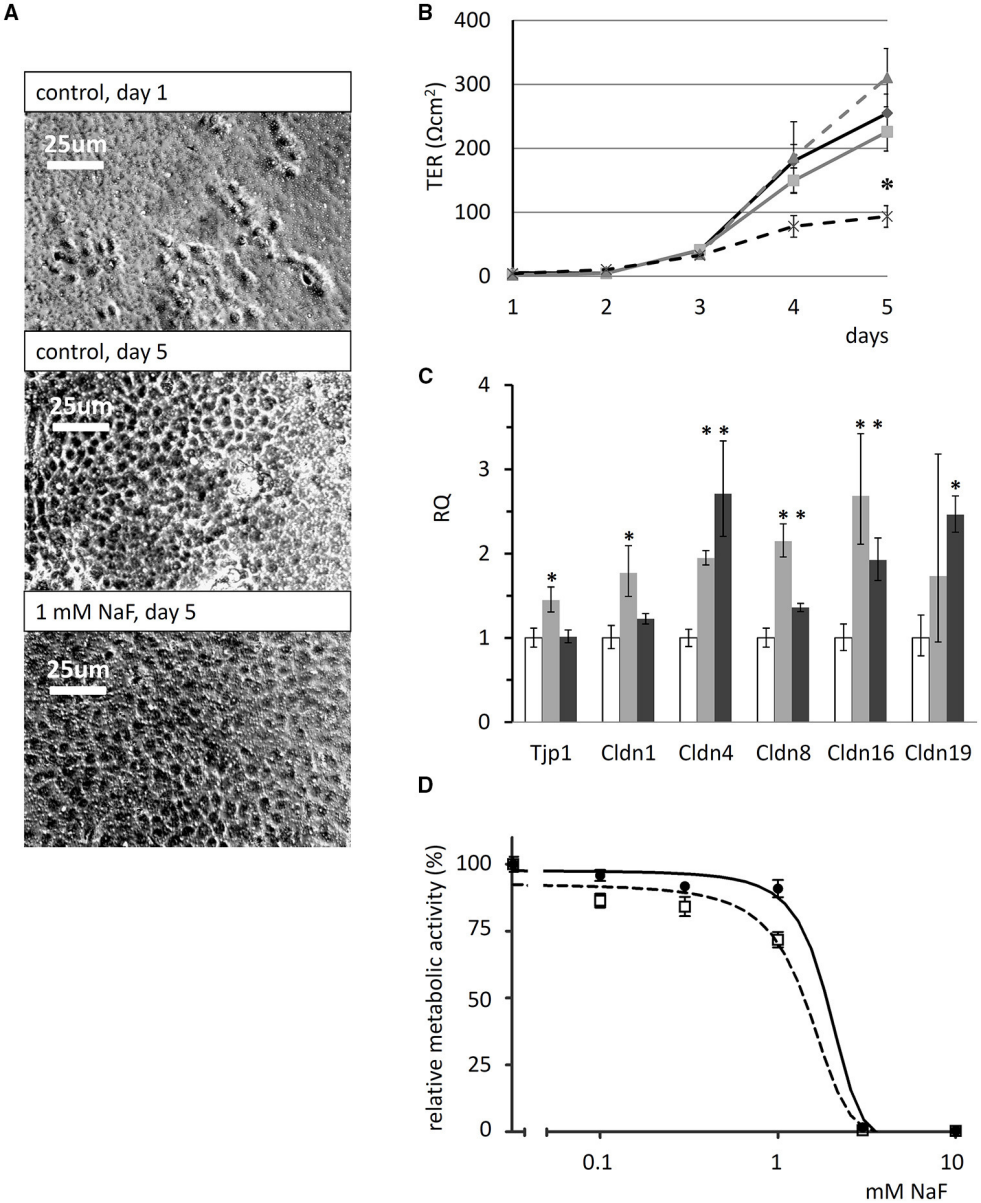
Effects of fluoride on transepithelial resistance, tight-junction protein expression and cell viability of HAT-7 cells**. (A)** Phase-contrast images of HAT-7 cells grown on Transwell membranes in control medium at day1 and 5 (control), or in the same medium supplemented with 1 mM NaF. **(B)** Transepithelial resistance (TER) of the cells cultured on Transwell membranes for 5 days in the absence (solid black) and presence of 0.3 mM (solid gray), 0.6 mM (broken gray), or 1 mM NaF (broken black line). A significant difference in TER was observed after 5 days when cells cultured with 1 mM NaF were compared to controls: ^*^p < 0.05. **(C)** Quantitative RT-PCR data showing expression of tight-junction genes Tjp1, Cldn1, Cldn4, Cldn8, Cldn16, and Cldn19 genes, normalized to mitochondrial Rplpo gene expression in HAT-7 cells treated as described above (*n* = 3 for each gene). Changes in gene expression following treatment with 0.6 mM (gray) and 1 mM (black) NaF are compared to controls (white): ^*^p < 0.05; error bars show 95% confidence intervals. **(D)** Concentration dependence of the effect of NaF on the metabolic activity of HAT-7 cells treated for 48 h (black circles, continuous line) and 96 h (empty rectangles, broken line) (*n* = 6 for each NaF concentration).

The formation of tight junctions is essential for ameloblast polarization and differentiation (Bartlett and Smith, 2013) and it creates an intercellular barrier that separates the apical and basolateral spaces, thus enabling transepithelial ion gradients to exist across the epithelium. We monitored tight-junction formation and polarization by measuring the transepithelial resistance (TER) of HAT-7 cells cultured on Transwell membranes for 5 days, performing daily TER measurements while the cells were exposed to various concentrations of NaF. The cells became confluent, covering the whole surface of the Transwell membranes, after 3–5 days in the presence of fluoride at concentrations up to 1 mM (phase-contrast images in Figure 6A). Over the 5-day period TER development was not significantly affected the by presence of 0.3 or 0.6 mM NaF. However, we detected an almost full inhibition of TER development by 1 mM NaF (*p* < 0.05 vs. zero fluoride control, Figure 6B).

Tight-junction forming gene expression in HAT-7 cells cultured on Transwell membranes in differentiation medium was evaluated by quantitative RT-PCR (Figure 6C). Unexpectedly, fluoride exposure did not inhibit the expression of the junctional complex genes Tjp1, Cldn1, Cldn4, Cldn8, Cldn16, and Cldn19 at mRNA level at all. Instead, a moderate but significant increase was observed in their expression. These data suggest that fluoride impedes tight junction assembly, rather than the expression of its key protein components.

The cytotoxicity of NaF was determined using the alamarBlue viability assay and this showed that the metabolic activity of HAT-7 cells was not altered by NaF concentrations of up to 0.6 mM (Figure 6D). With 1 mM fluoride, the concentration that impeded tight junction formation, metabolic activity was only slightly reduced. However, cell viability was preserved, as judged by the photomicrographs taken at day 5, which show full confluency of the cells (Figure 6A). In contrast, 3 mM NaF was totally toxic, killing the cells after just 48 h (Figure 6D).

## Discussion

The CO_2_/${\text{HCO}}_{3}^{-}$ equilibrium is central to the proper regulation of extracellular pH by ameloblasts during enamel mineralization (Lacruz et al., 2010, 2012, 2013; Bronckers et al., 2016). A major finding in our previous work was that HAT-7 cells grown as a monolayer on Transwell membranes are capable of apical-to-basolateral ${\text{HCO}}_{3}^{-}$ secretion (Bori et al., 2016). To identify the acid/base transporters responsible for basolateral ${\text{HCO}}_{3}^{-}$ accumulation in the cytosol during secretion, we first examined the recovery of pH_i_ following an acid load in the absence of ${\text{HCO}}_{3}^{-}$/CO_2_. This was dependent on basolateral Na^+^ and was almost completely blocked by basolateral application of amiloride, suggesting the presence of NHE1 at this membrane. This is consistent with the observation that the presence of a basolateral Na^+^/H^+^ exchanger, usually NHE1, is an almost universal feature of the epithelial cells of the gastrointestinal tract (Kiela et al., 2006). Under physiological conditions, in the presence of ${\text{HCO}}_{3}^{-}$/CO_2_, NHE1 contributes to bicarbonate accumulation within the cells, because it shifts the carbonic-anhydrase catalyzed reaction toward the production of ${\text{HCO}}_{3}^{-}$ ions by removing H^+^ from the cell. The importance of this mechanism can be clearly seen in other ${\text{HCO}}_{3}^{-}$-secreting epithelia such as those of the salivary glands and pancreas (Steward et al., 2005).

Besides H^+^ extrusion, Na^+^-${\text{HCO}}_{3}^{-}$ co-transporters (NBCs) may also contribute to ${\text{HCO}}_{3}^{-}$ uptake. In our study the presence of a basolateral Na^+^-${\text{HCO}}_{3}^{-}$ cotransporter was revealed in acid-loading experiments performed in the presence of ${\text{HCO}}_{3}^{-}$/CO_2_. The recovery of pH_i_ was Na^+^ dependent, and was only partially inhibited by amiloride. The simultaneous application of amiloride and H_2_DIDS resulted in a significantly greater inhibition suggesting that a basolateral NBC also contributes to the cytosolic ${\text{HCO}}_{3}^{-}$ supply. Moreover, when NHE activity was measured in ${\text{HCO}}_{3}^{-}$-free (HEPES-buffered) medium, the pHi recovery from acidosis (control) and its inhibition by NHE inhibitor was substantially lower than recovery rate and its inhibition by the NHE inhibitor in ${\text{HCO}}_{3}^{-}$-containing medium, further suggesting the existence of an NHE-independent mechanism. These data are consistent both with our RT-PCR evidence for NBCe1 expression in HAT-7 cells (Bori et al., 2016) and with previous reports of tissue staining in mid-maturation ameloblasts (Jalali et al., 2015). The basolateral localization of NBCe1 in these cells is similar to that observed in secretory epithelia in rat (Zhao et al., 1994) and guinea-pig (Ishiguro et al., 2000) pancreatic ducts, and also in rat (Gresz et al., 2002), and guinea-pig (Li et al., 2006) salivary glands.

Chloride ions are usually required for ${\text{HCO}}_{3}^{-}$ transport in secretory epithelia (Demeter et al., 2009a), and they are most likely also essential in pH modulation during enamel formation (Bronckers, 2017). There is a strong positive correlation between calcium content and chloride content during ongoing enamel maturation and ameloblast modulation. Lower than normal Cl^−^ content leads to hypomineralization (Bronckers et al., 2015). CFTR-null and AE2-null mice show strongly affected phenotypes in their enamel structure (Sui et al., 2003; Bronckers et al., 2015). Importantly, cells have to first accumulate Cl^−^ intracellularly in order to secrete it across the apical membrane. NKCCs are electroneutral symporters that move Na^+^, K^+^, and Cl^−^ ions into the cell by secondary active transport. NKCC activity can be detected by microfluorometry because of its ability to carry ${\text{NH}}_{4}^{+}$ ions in place of K^+^ (Paulais and Turner, 1992b). In this study, we observed a bumetanide-sensitive decrease in pH_i_ during NH_4_Cl exposure in HAT-7 cells. Therefore, the cotransporter (most probably NKCC1) may be an important contributor to Cl^−^ uptake across the basolateral membrane of HAT-7 ameloblast cells, as it is in a number of other secretory epithelia including the pancreatic ductal cell lines Capan-1 and HPAF (Szucs et al., 2006; Demeter et al., 2009a) and salivary acinar cell line Par-C10 (Demeter et al., 2009b), where Cl^−^ secretion is largely dependent on basolateral NKCC1 activity (Paulais and Turner, 1992a; Melvin et al., 2005). Our study represents the first functional evidence that NKCC1 could have a role in Cl^−^ accumulation in ameloblasts. Furthermore, it is in line with the recent observation that NKCC1 is expressed during amelogenesis in papillary cells by immunohistochemistry (Jalali et al., 2017).

Another major class of ${\text{HCO}}_{3}^{-}$ transporters are the anion exchangers (AEs). The Na^+^-independent AEs of the SLC4 family accomplish the electroneutral exchange of Cl^−^ with ${\text{HCO}}_{3}^{-}$ ions. According to our Cl^−^ substitution experiments, a Cl^−^/${\text{HCO}}_{3}^{-}$ exchanger is present at the basolateral membrane of HAT-7 cells, as confirmed by the inhibitory effect of DIDS. This is most likely to be the AE2 exchanger, whose expression we detected previously in polarized HAT-7 cells by immunocytochemistry (Bori et al., 2016) and which is expressed at the basolateral membranes of most epithelial cells (Romero et al., 2004). In salivary acinar cells, the basolateral Cl^−^/${\text{HCO}}_{3}^{-}$ exchanger provides an important additional pathway for the accumulation of intracellular Cl^−^ against its electrochemical gradient (Melvin et al., 2005; Demeter et al., 2009b). This basolateral location in HAT-7 cells is also consistent with previous reports of the basolateral expression of AE2 in maturation ameloblasts (Lyaruu et al., 2008, 2014).

A high level of fluoride exposure is known to impair enamel formation and can result in hypomineralization (Denbesten et al., 1985; Smith et al., 1993; Bronckers et al., 2009). The exact mechanism is unknown and multiple factors might contribute to this phenomenon. Fluoride may affect ion secretion by ameloblasts, the developmental and functional states of ameloblasts unrelated to ion secretion, and it could also contribute to the physical events of mineralization. To test the first possibility, we investigated how fluoride exposure affects transcellular ${\text{HCO}}_{3}^{-}$ secretion in HAT-7 cells. We have recently demonstrated that HAT-7 cells can accumulate ${\text{HCO}}_{3}^{-}$ ions through the basolateral membrane and in turn secrete them through the apical membrane (Bori et al., 2016). Our present data clearly show that acute exposure to a wide range of fluoride concentrations causes no change in the rate of acidification of the cells when basolateral ${\text{HCO}}_{3}^{-}$ uptake is blocked. These data indicate that fluoride has no acute inhibitory effect on ${\text{HCO}}_{3}^{-}$ secretion, which we consider to be a crucial requirement for mineralization (Varga et al., 2015).

The investigation of the effect of fluoride on ameloblast monolayer formation and function yielded interesting, and somewhat unexpected results. In our hands fluoride application up to 1 mM resulted in no, or very little, change in HAT-7 cell viability. However, increasing the fluoride concentration to 3 mM resulted in an almost complete loss of the cells, independent of the exposure period (2–5 days). Our findings are consistent with recent observations on HAT-7 cells by other investigators (Zhang et al., 2016) and with studies of the mouse LS8 ameloblast cell line (Kubota et al., 2005; Zhang et al., 2006, 2007; Sharma et al., 2008). Collectively, these studies suggest that ameloblast survival is not seriously affected up to millimolar concentrations, but further increases in the fluoride concentration result in rapid deterioration over a very narrow concentration range.

When we studied the effects of fluoride on the development of transepithelial resistance, we found substantially delayed TER development in doses below those producing cytotoxic levels. We hypothesized that the delay in tight-junction formation might be a consequence of changes in the expression of one or more tight-junction proteins. Thus, we investigated the expressional changes in Tjp1, Cldn1, Cldn4, Cldn8. Importantly, we have shown previously that the expression profiles of these proteins show some relationship with the normal development of TER in HAT-7 cells (Bori et al., 2016). We also evaluated the expression of Cldn16 and Cldn19, since their crucial role in ameloblast tight-junction formation has been recently indicated; their mutation causing familial hypomagnesaemia with hypercalciuria and nephrocalcinosis and amelogenesis imperfecta (Bardet et al., 2016; Yamaguti et al., 2017). To our surprise, fluoride exposure did not inhibit the expression of junctional-complex protein genes Tjp1, Cldn1, Cldn4, Cldn8, Cldn16, and Cldn19 at all. Instead, a moderate but significant increase was observed in their expression. These data suggest that fluoride impedes tight-junction assembly, rather than the expression of its protein constituents. Regarding the mechanism, several types of signaling pathways and proteins have been linked to tight junction assembly. The fluoride-sensitive RhoA-ROCK signaling is crucial in controlling epithelial polarity and adhesion of ameloblasts (Otsu and Harada, 2016), also directly regulating E-cadherin expression (Xue et al., 2013) which is fundamental for tight junction formation (Matter and Balda, 2003). Further studies will determine whether these elements are really linked together. This is particularly important, since the amelogenesis stage mimicked by the proposed polarized HAT-7 model should be better characterized in the future, particularly with respect of tight junctions at protein levels as the lack of this is a major limitation of the present study.

Our present findings indicating delayed tight-junction assembly might offer an alternative, or additional, explanation for dental fluorosis. Our data do not diminish the importance of the many other postulated mechanisms, such as delayed removal of matrix proteins in fluorosed maturation enamel (Denbesten et al., 1985; Smith et al., 1993), increased binding of amelogenins to fluoride-containing hydroxyapatite crystals (Tanimoto et al., 2008), reduced KLK4 expression by ameloblasts (Suzuki et al., 2014), increased SATB1 protein content and enhanced Gαq activity (Zhang et al., 2014), decreased trafficking of NCKX4 Ca^2+^-transporter to the apical membrane (Bronckers et al., 2017). To some extent, many or all of these mechanisms may contribute to hypomineralization depending on the actual local concentrations of fluoride. Nonetheless, the delay in tight-junction formation, could also be very important when one considers the structural and functional cycling of ameloblasts. Ruffle-ended ameloblasts cyclically turn into smooth-ended ameloblasts and vice versa during amelogenesis (Smith, 1998; Josephsen et al., 2010). In rats, a cycle lasts about 8 h, during which the cells are in the ruffle-ended state for about 4 h before abruptly changing to the smooth-ended phenotype for about 2 h. Afterwards, the ruffled border of the cell membrane facing the enamel is gradually rebuilt and the tight junctions are translocated and reassembled (Smith, 1998; Josephsen et al., 2010). If the disassembly and reassembly of tight junctions is as important as this model suggests, any delay in their turnover could have serious consequences for the amelogenesis process itself. Since the present observations were obtained in an *in vitro* cellular model, our hypothesis is only tentative, but we certainly believe that it deserves further investigation. However, we have to state that the polarized HAT-7 model needs to be further characterized, and other cellular models, including human ameloblast models have to be developed to support the validity of the above proposed hypothesis.

**Figure 7:**
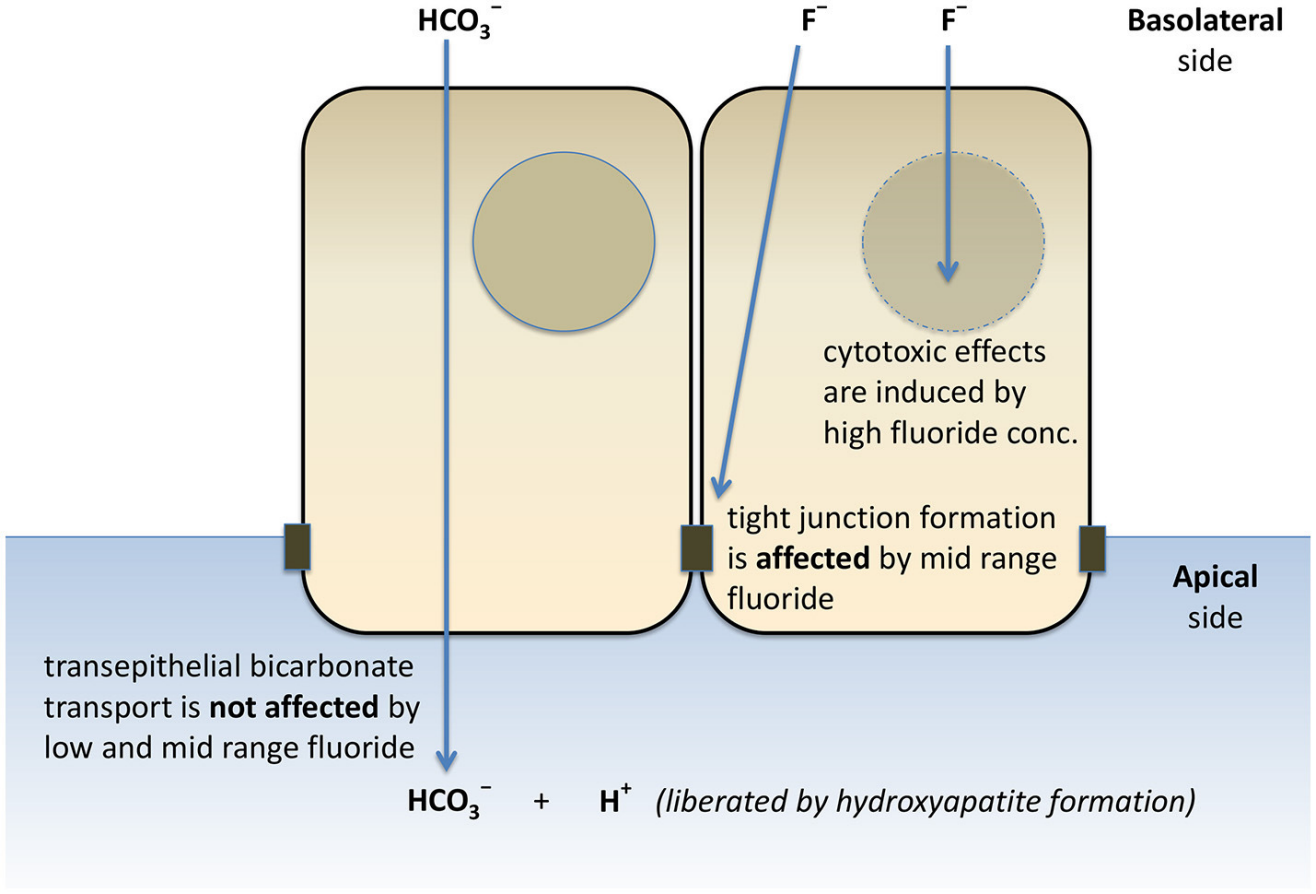
Schematic representation of the effects of fluoride on ${\text{HCO}}_{3}^{-}$ secretion, tight junction formation, and cell death of HAT-7 cells. Bilateral exposure of 2D monolayers of HAT-7 cells differentiated on Transwell membranes to low-range (0–50 μM), mid-range (100–1,000 μM) fluoride and high fluoride concentration (3 mM) resulted in differential changes in ameloblast function.

In conclusion, our HAT-7 model is a useful tool for the functional analysis of ameloblast pH regulation and the associated ion transport mechanisms. We have verified the activity of several key transporters affecting the pH regulation and vectorial ${\text{HCO}}_{3}^{-}$ and Cl^−^ transport by these cells. Furthermore, we have provided evidence that ${\text{HCO}}_{3}^{-}$ secretion is not affected by a wide range of fluoride concentrations. However, the formation of tight junctions is severely delayed by 1 mM fluoride, a concentration which does not have substantial cytotoxic effects (Figure 7). This hitherto unknown effect of fluoride may prove to be an important factor in the development of dental fluorosis.

## Author contributions

RR: contributed to conception and design, data acquisition, analysis, and interpretation, drafted and critically revised manuscript; AF: contributed to data acquisition, analysis, and interpretation, and critically revised manuscript; EB: contributed to conception and design, data acquisition, analysis, and interpretation, and critically revised manuscript; ÁZ and GG: contributed to data analysis and interpretation, and critically revised manuscript; HH: contributed to conception and to data interpretation, and critically revised manuscript; MS: contributed to design, data analysis and interpretation, and critically revised manuscript; PD: contributed to conception and design, data interpretation, and critically revised manuscript; AB: contributed to conception, design, data analysis, and interpretation, and drafted and critically revised manuscript; GV: contributed to conception, design, data analysis and interpretation, and drafted and critically revised the manuscript.

### Conflict of interest statement

The authors declare that the research was conducted in the absence of any commercial or financial relationships that could be construed as a potential conflict of interest. The reviewer CB and handling Editor declared their shared affiliation.
